# A Report of Two Cases of Age-Related Changes in Cervical Morphology in Postmenopausal Women with Vaginal Adenosis

**DOI:** 10.1155/2017/9523853

**Published:** 2017-01-02

**Authors:** Marguerite B. Vigliani

**Affiliations:** Alpert Medical School at Brown University, Providence, RI, USA

## Abstract

This paper presents two cases of women who had extensive vaginal adenosis from prenatal DES exposure, extending almost halfway down the vaginal canal. Both women were followed for decades with annual exams and Pap smears until after menopause. Clinical examination in both cases initially showed an absent pars vaginalis of the cervix, vaginal adenosis, and shallowness of the fornices. Several decades of annual exams showed these stigmata of DES exposure gradually disappear as the upper vagina progressively contracted. After menopause the upper vagina in both cases transformed into what appeared to be a normal cervix with all adenosis involuted into a normal endocervical canal. A timeline was created to show the morphological changes that were observed over time. This timeline illustrates how severe vaginal stenosis above the level of the squamocolumnar junction developed in middle age and was followed in the postmenopause by fusion of the upper vaginal walls in the midline resulting in the appearance of a normal, but prolapsed, cervix.

## 1. Introduction

Structural abnormalities of the cervix and upper vagina associated with prenatal DES exposure have been described by authors who examined enrollees in the National Cooperative Diethylstilbestrol Adenosis Project (DESAD). The described abnormalities include absent pars vaginalis of the cervix, hypoplastic cervix, transverse cervical and vaginal ridges, absent vaginal fornices, and various anterior cervical lip protuberances called cervical ridges, collars, cockscomb deformities, hoods, rims, and pseudopolyps [1–5]. In general, these structural and epithelial changes have been reported as slowly, partially, or completely resolving with time [6] but there is limited information about long-term followup because DESAD examined enrollees for only 5 years. At the end of the project in 1984 many of the enrolled DES-exposed women were still young.

In contrast, this report details decades of observations on two patients with DES-associated vaginal adenosis, yielding some interesting longitudinal observations. In both cases the upper vaginal walls contracted and lost their elasticity progressively during the reproductive years, making it increasingly difficult to visualize the cervix. Complete resolution of DES-associated epithelial and structural changes did not occur until well after menopause, much longer than what has previously been described.

## 2. Case 1

The patient was a white female who visited the office for annual exams and Pap smears because of a history of prenatal DES exposure. Her gynecological history included preterm labor, premature delivery, growth retardation, and fetal distress. In her 30s and 40s, she had postcoital and intermenstrual bleeding, but workups including colposcopy showed only vaginal adenosis without dysplasia.

During the reproductive years there was no palpable or visible cervix. The cervical os appeared as a pinpoint aperture in a flattened, shallow upper vagina, bearing patchy red islands of glandular epithelium, or vaginal adenosis. Each year the upper vagina contracted and visualization of the cervical os became increasingly difficult. The patchy erythema became patchy leukoplakia. She experienced menopause at age of 49.

**Figure 1:**
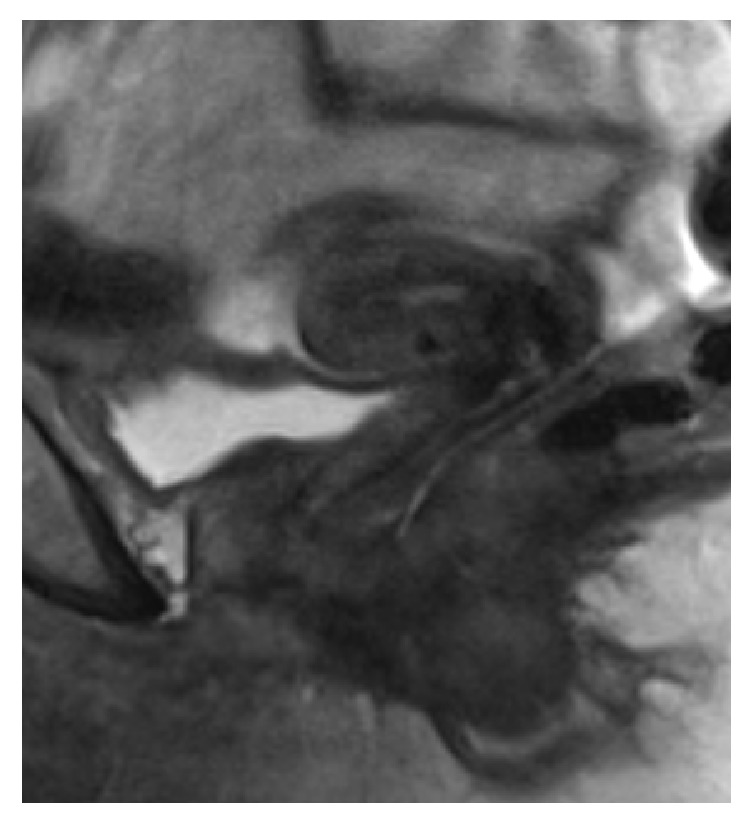
Sagittal MRI in Case 1 showing uterus, cervix, and vagina after menopause.

After menopause an extra-narrow Pederson speculum could not be opened completely in the upper vaginal canal because of narrowing and fibrosis. Pap smears were obtained by blindly twirling a dacron urethral swab inside the tightly constricted upper vaginal tunnel. The patient was offered vaginal estrogen to open the upper vagina, but she declined. Eventually, the cone-shaped narrow upper vagina closed completely and examination showed what appeared to be a prolapsed cervix located midway down the vagina. An MRI confirmed the presence of a normal, prolapsed cervix (Figure 1).

**Figure 2:**
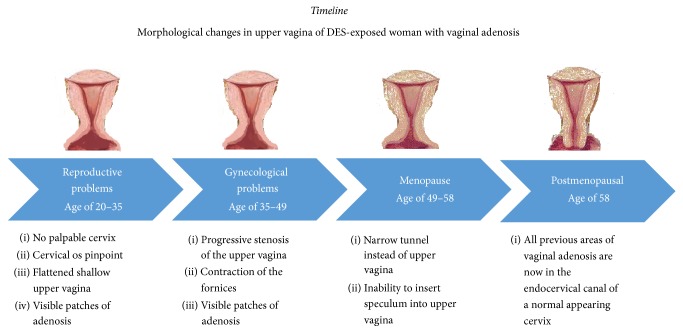
Timeline for Case 1 with schematic diagram showing the authors' impression of progressive contraction of upper vagina with involution of vaginal adenosis into a new cervix.

A timeline (Figure 2) illustrates the author's impression of clinical changes over time.

## 3. Case 2

The patient was a white female who gave a history of prenatal DES exposure. Here menses were irregular and she had androgen excess, diabetes, hypertension, and morbid obesity. Two of her three pregnancies required induction of ovulation with clomiphene. The first pregnancy ended at sixteen weeks because of cervical incompetence. Her two subsequent pregnancies were both managed with prophylactic cerclage. The first of these was a twin gestation delivered by Cesarean, and the second was a singleton pregnancy delivered vaginally after removal of the cerclage.

During her early reproductive years the cervix was flat and hypoplastic. By age of 36 the upper vagina had assumed a cone shape and the cervix became difficult to visualize. After her last pregnancy the vaginal sidewalls converged at the apex and the cervix appeared to be a dimple in the upper vagina. This dimple was difficult to see as it was very deeply located in a narrow upper vaginal tunnel. Neither a diaphragm nor a cervical cap could be fitted because of the anatomical distortion of the upper vagina. Her Pap smears were mildly atypical and colposcopic biopsies confirmed CIN I, which was managed expectantly.

**Figure 3:**
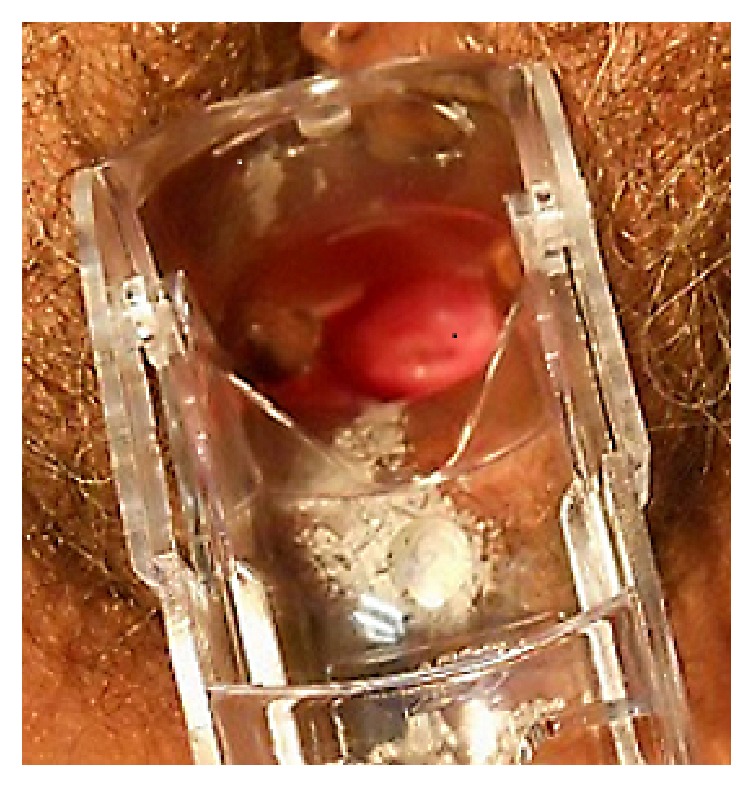
Photograph of Case 2, showing what appears to be a cervix in a postmenopausal woman who had undergone a total hysterectomy with bilateral salpingo-oophorectomy.

At age of 48 she was diagnosed with hormone positive breast cancer and was treated with lumpectomy, radiation, and chemotherapy. Her menses ceased during treatment. Subsequently she received adjuvant hormone therapy with an aromatase inhibitor. A few years later a total abdominal hysterectomy and bilateral salpingo-oophorectomy was performed because of a 10 cm adnexal mass. On pathology the mass was a benign ovarian teratoma with struma ovarii. The pathology report also described the uterus, the fallopian tubes, the contralateral ovary, and a cervix comprised of an ectocervix, described as being pink-red, hemorrhagic, and measuring 1.5 × 2.0 with a 0.8 cm slit-like os with an endocervical canal that was cystic, yellow-pink, and measured 3.0 × 1.0 cm. In the endocervical canal there was a clear, fluid-filled cyst measuring 1.8 cm in diameter. This cyst was identified as a deep nabothian cyst. Despite gross and histological confirmation that the cervix had indeed been removed, a year later pelvic examination showed what appeared to be a cervix-like structure with an os that could be cannulated and sampled with an endobrush. This neocervix was located partway down the vaginal canal as if it were prolapsed (Figure 3).

## 4. Discussion

These two cases differ somewhat in that Case 1 experienced a natural menopause and it took a few years after cessation of menses for complete resolution of her DES-associated adenosis. Case 2 experienced cessation of menses induced by chemotherapy and hormone therapy with an aromatase inhibitor. Surprisingly, her DES-associated adenosis did not resolve until she had bilateral oophorectomy several years later, indicating perhaps that the continued presence of estrogens or androgens from the postmenopausal ovary in some way prevented complete resolution of DES-associated vaginal adenosis by a pathway not blocked by the aromatase inhibitor.

One observation common to both cases is that the ectopic glandular epithelium in the upper vagina eventually involuted into what appeared to be a normal endocervical canal as the vaginal walls thickened, fibrosed, and fused to form a cervix-like structure. This observation is consistent with the idea that it is not epithelial migration, but rather stromal or mesodermal transformation that determines the morphology of the cervix and vagina.

Recent research with animal models as well as in vitro experiments have explored the various roles of gene expression, immunohistochemistry, and embryological induction phenomena in the development of the vagina and cervix in the embryo [7]. These studies with animal models point to the idea that Mullerian-derived mesenchyme is identified early on in development as being as either uterine mesenchyme or vaginal mesenchyme. These two cases, which show postmenopausal resumption of DES-disrupted mesodermal embryological development, support the idea that the stromal tissue underlying vaginal adenosis thought to be the cranial portion of the vagina is really uterine mesenchyme in origin.

## 5. Conclusion

Consideration of the morphological changes that occurred in these two cases highlights the importance of continuing to sample the upper vagina and cervix for Pap smears in elderly women who had been exposed to prenatal DES, even if sampling becomes very difficult or even if there was a hysterectomy with the cervix removed. Clinicians should remember that the risk of clear-cell adenocarcinoma might still be present in DES-exposed women due to the exposure of ectopic epithelium in the vagina during reproductive years with possible exposure to carcinogens. This reminder is especially relevant for younger clinicians who may be less familiar with the structural and epithelial changes associated with prenatal DES exposure.
